# Special Issue “Molecular Biology in Forensic Science: Past, Present and Future”

**DOI:** 10.3390/ijms25052883

**Published:** 2024-03-01

**Authors:** Francesco Sessa, Monica Salerno

**Affiliations:** Department of Medical, Surgical and Advanced Technologies “G.F. Ingrassia”, University of Catania, 95121 Catania, Italy; monica.salerno@unict.it

## 1. Introduction

Molecular biology has always represented an enviable tool in the fields of biosciences, diagnostics, and forensic sciences. Its importance is constantly emphasized not only within individual disciplines but also in their interconnectedness. In recent years, the speed with which molecular biology techniques have enormously improved in the forensic field has shown that their impact can extend beyond the boundaries of forensics, affecting neighboring areas such as diagnostics. The rapid development of forensic molecular biology has transformed into a comprehensive discipline, leaving a deep imprint that is now evident not only in the field of forensic genetics [1,2]. As described in the literature, the integration of molecular biology technologies into forensic science has facilitated advances in criminal investigations, paternity disputes, the identification of missing persons, and exoneration of the wrongfully convicted. In addition, forensic molecular biology has extended its reach into areas such as forensic pathology, anthropology, and wildlife forensics, increasing capabilities in species identification, population genetics, and conservation efforts [3,4]. In particular, the use of increasingly advanced technologies has also enabled the resolution of “cold cases”, although numerous pitfalls remain related to the increased sensitivity of these techniques, such as “touch DNA”, “direct transfer”, and “indirect transfer” [5,6,7,8,9].

Concerning forensic DNA analysis, molecular biology has undoubtedly undergone rapid changes in recent decades. Indeed, it should not be forgotten that the first investigations in this field started with the assessment of restriction fragment length polymorphisms (RFLPs), while today methods have become increasingly sensitive and specific, moving from gel electrophoresis techniques to capillary electrophoresis and finally to next-generation sequencing (NGS) [10,11,12]. NGS represents a new frontier in genetic analysis technology, offering unprecedented potential, that aims to become the gold standard for genetic analysis in forensic applications. With its ability to process massive amounts of DNA data at unprecedented speeds, NGS has redefined the forensic molecular biology landscape while also providing new perspectives in the field of diagnostics, improving DNA profiling and forensic identification capabilities.

Looking ahead, the evolution of forensic molecular biology is poised to profoundly shape the future of forensic science and diagnostic fields. As technologies continue to advance, the convergence of NGS, microRNA (miRNA) analysis, and other modern molecular biology tools will pave the way for advanced forensic investigations, artificial intelligence applied to the forensic field, and personalized diagnostics. Thus, the future looks promising not only regarding rapid advancements but also improving the reliability of investigations in forensic cases and in the field of public health [6,12,13,14,15].

In this area, for example, the exploration of miRNA is a compelling field of research with implications for both forensic science and health care. miRNAs, small noncoding RNA molecules, play a key role in post-transcriptional gene regulation, influencing various cellular processes. Their potential relevance in forensic molecular biology stems from their stability in biological samples even in the post-mortem period, with the possibility of testing them even on fixed samples included in paraffin. All this suggests their possible use as biomarkers not only for forensic investigations, such as in the determination of the “time since death”, but also as possible theranostic markers. miRNAs show distinctive expression patterns in response to various physiological and pathological conditions, making them potential markers as much in forensic diagnostics and medicolegal investigations as in clinical practice. By expanding our knowledge in this area, expression analyses of different miRNAs may lead to the identification of new markers for tissue changes related to aging and/or pathophysiological conditions, thereby strengthening the scope of forensic science research [15,16,17]. Indeed, in recent years, data sharing between seemingly distant fields such as forensic science and clinical practice has facilitated the cross-pollination of knowledge, leading to advances in both fields [12,18,19].

In this context, this Special Issue aimed to collect accurate and up-to-date scientific information on all aspects of forensic molecular biology techniques and applications that are currently used for the analysis of human and non-human samples in the form of original research articles, case series, case reports, and reviews.

## 2. Special Issue Manuscripts

This Special Issue comprises ten articles (see Table 1), consisting of seven original research studies and two reviews, to which 61 authors have contributed. Several other papers that were submitted did not pass the stringent quality criteria that we set.

As summarized in Table 1, the most challenging research field in forensic sciences is related to the determination of the post-mortem interval (PMI): indeed, 3/10 articles in this Special Issue investigated this theme. Marrone et al. (contribution 1) investigated early post-mortem changes in the proteome profile of pig skeletal muscle to identify potential PMI biomarkers. Through mass spectrometry-based proteomics, nine potential PMI biomarkers were identified, with their quantities changing progressively over time. The study emphasizes the significance of the proteomic approach in developing a reliable method for PMI determination and the need to characterize a large number of marker proteins for forensic practice. On the same theme, Rubio et al. (contribution 2) presented a study that aimed to assess the correlation between changes in the mineral composition of human teeth and the estimation of the PMI. The results showed that certain parameters, such as the crystallinity index, crystal size index, mineral-to-organic matrix ratio (M/M), and carbonate/phosphate ratio (C/P), had a strong association with the PMI, suggesting that these parameters could be highly accurate in its estimation. Furthermore, Bianchi et al. (contribution 3) discussed a pilot study that aimed to assess the feasibility of using NGS analysis on dental pulp to detect genetic mutations in DNA caused by post-mortem cell necrosis. The authors concluded that 38 out of the 56 considered genes were affected by mutations, with 14 mutations occurring only within specific ranges of PMIs/ADD (Accumulated Degree Days). These authors demonstrated that NGS analysis on dental DNA could be considered promising to estimate PMIs of several days.

Two articles (one experiment and one Review) focused on the importance of the sampling method in forensic genetics. In particular, Tozzo et al. (contribution 4) discussed the importance of collecting and interpreting “touch DNA” from crime scenes. This literature review compared three different sampling procedures—“single-swab”, “double-swab”, and “other methods” (including cutting out, adhesive tape, and FTA^®^ paper scraping)—concluding that the single-swab method was more efficient in DNA recovery across a wide variety of experimental settings compared with the double-swab technique and other methods. Moreover, in their research Zapico et al. (contribution 5) aimed to evaluate the efficiency of different swab types for body fluid identification and DNA isolation and characterization. The study provided an analysis of the impact of swab types on body fluid identification using immunochromatographic tests, and it supports previous research findings related to the influence of swab types on nuclear DNA isolation and characterization.

Onofri et al. (contribution 6) published a study focused on standardizing and optimizing a DNA methylation-based protocol for age prediction in forensic contexts, tailored to the Italian population. This study outlined precise steps, including DNA extraction, bisulfite conversion, amplification, purification, single base extension, capillary electrophoresis, and result analysis, to train and test the tool. The obtained prediction error showed a mean absolute deviation of 3.12 years in the training set and 3.01 years in the test set, suggesting that further improvements could be achieved by implementing additional samples representative of the entire Italian population. The same research group (contribution 9) described the possibility of recovering a DNA profile after a transfer, sampling a personal item in situations where the owner and person of interest share the same workspace. It also investigates secondary and higher degree transfer scenarios of non-self-DNA deposition. The study emphasizes the importance of collecting information about DNA transfer probabilities and the presence of the person of interest in the ‘baseline’ bgDNA of the substrates involved.

Sessa et al. (contribution 7) introduced the use of a forensic biobank for translational medicine. In particular, these authors discussed the role of miRNAs in regulating heart functions and their association with cardiovascular diseases (CVDs). They introduced the concept of theranoMiRNAs, which are miRNAs that may be used for both the diagnosis and treatment of heart diseases. The study focused on the use of bioinformatic tools to clarify miRNA interactions with candidate genes and emphasized the importance of a computational approach in establishing associations between miRNAs and target genes. The results elucidate the molecular mechanisms linking miRNAs to CVDs and highlight the importance of in vivo studies to obtain further evidence in this challenging field of research, especially considering that CVDs are the leading cause of death worldwide.

Maciejczyk et al. (contribution 8) focused on toxicological investigations, reporting the results of an experimental study that investigated the use of antioxidant and oxidative stress biomarkers in various post-mortem biological fluids of individuals who died from acute alcohol intoxication. The study found significant increases in certain antioxidant markers in urine but not in other circulating fluids. Additionally, no connection was observed between the oxidation–reduction balance and the amount of alcohol consumed before death. These authors concluded that the use of circulating body fluids to assess redox homeostasis in post-mortem analysis is limited, and further research is needed to understand the effects of post-mortem processes on cellular redox balance and the intensity of oxidative and carbonyl stress in ethanol-damaged organs.

Finally, in their Review Cardinali et al. (contribution 10) highlighted the importance of animal genomics in cases involving attacks, property destruction, or when non-human biological material is linked to a victim or perpetrator. While only a few animal genetic laboratories are able to conduct valid DNA profiling, the field of forensic science now focuses on analyzing genetic markers such as STRs and DNA SNPs from domestic species; this has also expanded to include wildlife. The use of these molecular markers in wildlife has become increasingly relevant for addressing illegal trafficking, protecting biodiversity, and conserving endangered species. Additionally, advancements in third-generation sequencing technologies have opened new possibilities for conducting forensic analysis directly in this challenging field.

## 3. Conclusions

The articles published in this Special Issue address several interesting and diverse aspects of the topic and offer new findings that contribute significantly to the advancement of molecular biology applications in forensic science.

In the future, there are still significant gaps to be filled in the field of genomics applied to forensic science. Future research efforts will play a key role in filling these gaps by improving existing techniques, increasing their applications, and exploring the potential of emerging technologies. In the forensic science context, however, there appears to be an increasing need to validate new applications by creating standardized protocols and guidelines.

In conclusion, the results presented in this Special Issue draw attention to the future challenges that the forensic science field must be prepared to meet.

## Figures and Tables

**Table 1:** Contributions to the Special Issue "Molecular Biology in Forensic Science: Past, Present and Future".

Authors and Country	Title	Topic	Type
Marrone el al., Italy (contribution 1)	Forensic Proteomics for the Discovery of New post mortem Interval Biomarkers: A Preliminary Study	PMI biomarkers	Original article
Rubio et al., Spain (contribution 2)	Partners in Postmortem Interval Estimation: X-ray Diffraction and Fourier Transform Spectroscopy	PMI biomarkers	Original article
Bianchi et al., Italy (contribution 3)	Dental DNA as an Indicator of Post-Mortem Interval (PMI): A Pilot Research	PMI biomarkers	Original article
Tozzo et al., Italy (contribution 4)	Touch DNA Sampling Methods: Efficacy Evaluation and Systematic Review	Sampling methods	Review article
Zapico et al., U.S.A. (contribution 5)	The Perfect Match: Assessment of Sample Collection Efficiency for Immunological and Molecular Findings in Different Types of Fabrics	Sampling methods	Original article
Onofri et al., Italy (contribution 6)	Forensic Age Estimation through a DNA Methylation-Based Age Prediction Model in the Italian Population: A Pilot Study	Forensic Age Estimation	Original article
Sessa et al., Italy (contribution 7)	New Insight into Mechanisms of Cardiovascular Diseases: An Integrative Analysis Approach to Identify TheranoMiRNAs	miRNA biomarkers	Original article
Maciejczyk et al., Poland (contribution 8)	Do Circulating Redox Biomarkers Have Diagnostic Significance in Alcohol-Intoxicated People?	Toxicology	Original article
Onofri et al., Italy (contribution 9)	Trace DNA transfer in co-working spaces: The importance of background DNA analysis	DNA background	Original article
Cardinali et al., Italy (contribution 10)	The Revolution of Animal Genomics in Forensic Sciences	Animal genomics	Review article
